# Prevalence and Multidrug Resistance Pattern of Methicillin Resistant *S. aureus* Isolated from Frozen Chicken Meat in Bangladesh

**DOI:** 10.3390/microorganisms9030636

**Published:** 2021-03-18

**Authors:** Mst. Sonia Parvin, Md. Yamin Ali, Sudipta Talukder, Azimun Nahar, Emdadul Haque Chowdhury, Md. Tanvir Rahman, Md. Taohidul Islam

**Affiliations:** 1 Population Medicine and AMR Laboratory, Department of Medicine, Faculty of Veterinary Science, Bangladesh Agricultural University, Mymensingh 2202, Bangladesh; soniaparvin@bau.edu.bd (M.S.P.); yamin2301@gmail.com (M.Y.A.); s.talukder39857@bau.edu.bd (S.T.); 2 Department of Livestock Services, Farmgate, Dhaka 1215, Bangladesh; 3 Department of Medicine, Faculty of Veterinary Science, Bangladesh Agricultural University, Mymensingh 2202, Bangladesh; azimunripa@gmail.com; 4 Department of Pathology, Faculty of Veterinary Science, Bangladesh Agricultural University, Mymensingh 2202, Bangladesh; emdad001@yahoo.com; 5 Department of Microbiology and Hygiene, Faculty of Veterinary Science, Bangladesh Agricultural University, Mymensingh 2202, Bangladesh; tanvirahman@bau.edu.bd

**Keywords:** methicillin-resistant *Staphylococcus aureus*, multidrug resistance, *mecA* gene, frozen chicken meat, Bangladesh

## Abstract

Infections by methicillin-resistant *Staphylococcus aureus* (MRSA) are continuously expanding within the community. Chicken meat is usually contaminated by MRSA, and this contaminated chicken meat is an important source of foodborne infections in humans. In this study, a cross-sectional supershop survey was conducted to determine the prevalence and antimicrobial resistance pattern of MRSA in 113 domestic frozen chicken meat samples purchased from nine branded supershops available in five divisional megacities of Bangladesh. The study also focused on the determination of methicillin resistance gene in MRSA isolates. *S. aureus* was identified by standard culture-based and molecular methods, and subjected to antimicrobial susceptibility testing. MRSA was screened by cefoxitin disk diffusion test. Methicillin resistance gene was identified by PCR. Of samples, 54.9% were positive for *S. aureus*, and, of these, 37.1% isolates were identified as MRSA. All the isolates were multidrug resistant (MDR): 52.2% were resistant to 6–8 antimicrobial classes, and 47.8% isolates to 9–12 classes. Three (3.2%) isolates of *S. aureus* were possible extensively drug resistant. The highest rates of resistance were observed against cefoxitin (100%), followed by nalidixic acid, ampicillin and oxacillin (97.7%), colistin (91.3%), amoxicillin-clavulanic acid and amoxicillin (87%), penicillin-G and cloxacillin (82.6%), oxytetracycline (78.3%), and cefixime (73.9%). Screening of methicillin resistance gene revealed that 43.5% isolates of MRSA were positive for *mecA* gene. The high prevalence of MDR MRSA in frozen chicken meat samples in this study emphasizes the need for better sanitary education of food handlers in hygienic practices focusing on their potential role as reservoirs and spreaders of MRSA.

## 1. Introduction

Foodborne diseases (FBDs) are a significant general well-being concern worldwide [1]. The World Health Organization (WHO) characterizes FBD as “disease of infectious or toxic nature caused by, or thought to be caused by, the consumption of food or water” [1]. *Staphylococcus aureus* (*S. aureus*) is one of the most important causes of FBD, causing an expected 241,000 illnesses each year in the United States [2]. *S. aureus* is an opportunistic pathogen in human and animals, and is the third largest cause of food related illness throughout the world [3,4]. *S. aureus* can cause a wide spectrum of infections, from superficial skin infections to severe, and probably fatal, invasive illness [5]. Ingestion of staphylococcal enterotoxins created in food by *S. aureus* enterotoxigenic strains results in staphylococcal food poisoning that can be thought of as perhaps the most widely recognized foodborne infection [3]. Among diverse kinds of foodstuffs, chicken meat handling with poor hygienic practice is exceptionally related with contamination of *S. aureus* enterotoxin [3]. Outbreaks can be added to numerous components including improper cooking, inadequate preparation of food, and contaminated water or raw materials used for food preparation [2].

Currently, *S. aureus* is a notorious bacterium, which quickly develops resistance to different antimicrobials [6]. The resistance is normally procured by horizontal gene transfer; although mutation and selection are also significant [7]. The most useful antimicrobials in the treatment of diseases brought about by *S. aureus* are β-lactams, including penicillin, methicillin, cloxacillin, oxacillin, flucloxacillin, and dicloxacillin [8]. Methicillin resistance in this bacterial species is very alarming for human wellbeing, as it has shown potential for zoonotic transmission [9]. Moreover, in step with the sensitivity to antibiotic medication, *S. aureus* was divided into methicillin-sensitive *Staphylococcus aureus* (MSSA) and methicillin-resistant *Staphylococcus aureus* (MRSA) [10]. In recent years, MRSA is attracting in depth attention. It usually showed multiple drug resistance (MDR) due to the abuse of antimicrobials for therapy and prophylaxis, and later these resistant MRSA enter into the food chains to cause human infections [11]. Thus, MRSA is recorded as one of 12 families of microorganisms that represent the greatest threat to human health [11]. The threat is probably equal or greater in developing countries like Bangladesh. Thus, the WHO recently classified MRSA as “high priority 2 pathogens” [6]. The magnitude of the general public health burden because of MDR in MRSA is complicated, and is influenced by variety of factors like antimicrobial use practices in farming, control measure at slaughter, storage and distribution systems, the supply of fresh water, and correct preparation and residential hygiene etc. [12].

The *mecA* gene, responsible for methicillin resistance in *S. aureus*, is the reason for these groups of microorganisms to be viewed as resistant to all beta-lactam antibiotics [13]. Methicillin resistance in *S. aureus* is mediated by the *mecA* gene, which encodes for a variant of penicillin-binding protein (PBP), PBP2a [13]. However, PBP2a has low affinity to methicillin [13]. The *mecA* gene, originally identified in MRSA, resides on a mobile genetic element, the staphylococcal cassette chromosome *mec* (SCC*mec*) that confers resistance to multiple antimicrobials [13]. SCC*mec* carries either the *mecA* or *mecC* gene, regulatory genes, or a variety of accessory genes encoding for a new specific PBP2a, which may carry additional AMR determinants in MRSA [14].

MRSA has been reported in a variety of meats including raw and frozen chicken, turkey, veal, beef, and mutton [10,15,16]. Detection of MRSA in frozen chicken meat has also been reported in some countries in different proportions, for example, China 8.1%, Hong Kong 7%, and Egypt 5.6% [10,11,17]. Chicken meat might be contaminated with MRSA during slaughtering or later during the meat preparation [10,11]. Contaminated chicken meat and meat products with MRSA is one amongst the main causes of digestive illness of humans in developing countries and will be counted collectively of the most important causes for morbidity and mortality [6]. The presence of MRSA in raw and frozen chicken meat can be considered as a marker for poor hygiene and improper storage conditions [10,11]. As it is well known that cooling and freezing temperatures can help improve safety as well as prolong the shelf life of chicken meat and meat products by delaying or inhibiting the growth of microorganisms, but *S. aureus*, especially MRSA, appear to be relatively resistant to the adverse effects of freezing [18]. In recent past, MRSA isolated from chicken meat samples were reported to be highly resistant to a number of multiple antimicrobials such as penicillin, methicillin, oxacillin, cefoxitin, chloramphenicol, and erythromycin that pose a big threat to the consumers’ health [6,10,11].

Recently, microbial food safety has obtained significant public wellbeing concern around the world including Bangladesh due to its huge effect on the food chain. Chicken meat had been widely consumed for its high value in protein and nutrient substance including low cost and availability. However, chicken meat is usually contaminated by antibiotic resistant strains of *S. aureus*, especially MRSA, mostly due to improper handling by food-handlers and poor sanitation practices, and thus poses a great risk in the food safety [12]. Contaminated chicken meat is one of the significant causes of FBDs in humans worldwide. Research has been conducted in Bangladesh on *S. aureus* contamination and AMR patterns in raw chicken meat from live bird markets [19,20,21]. Nowadays, city dwellers of Bangladeshi consumers prefer to buy their all essential daily commodities including frozen chicken meat and meat products from supershops because of easy availability, and this frozen chicken meat takes minimal processing for cooking, and also saves consumers time. However, contamination of frozen chicken meat with *S. aureus*, especially MRSA, has an important concern of food safety and public health hazards in Bangladesh. One inland study reported *S. aureus* contamination in preprocessed raw chicken meat collected from three supershops of Dhaka city [22], and another study reported the contamination of processed raw meat with MRSA [23]. However, prevalence and MDR pattern of MRSA as well as detection of methicillin resistance gene isolated from frozen chicken meat samples have not been investigated thoroughly and are not well documented in Bangladesh. Therefore, particular attention is required to be paid regarding studying the prevalence and AMR patterns of MRSA isolated from frozen chicken meat covering more outlets of available branded supershops to take effective measures in limiting the contamination of frozen chicken meat with MRSA and to protect consumers’ health. Therefore, the objective of the present study was to determine the prevalence of MRSA and their AMR pattern isolated from frozen chicken meat of different supershops across different divisional megacities in Bangladesh. This study also focused on the determination of methicillin resistance gene in MRSA isolates. Such information is useful for better understanding of the risk of exposure to MRSA through food, particularly chicken meat.

## 2. Materials and Methods

### 2.1. Sample Collection

From April to December 2019, a cross-sectional supershop survey was conducted in 40 outlets of nine different supershops in five divisional megacities (Dhaka, Chattogram, Sylhet, Rajshahi, and Mymensingh) of Bangladesh. A total of 113 domestic frozen chicken meat samples (82 broilers, 31 cockerels) including whole chicken or meat cuts (breast, drumstick, leg, and wing muscle) were purchased from these outlets. Each supershop had its own branded frozen chicken meat. On purchase, each sample was placed in a separate sterile tightly sealed plastic bag and kept in a cold box at a temperature lower than 4 °C for transportation. On arrival at the laboratory, frozen chicken meat in the sealed plastic bag was placed in a cool area until it defrosted. In addition, data on brand name, source of chicken, processing and packaging of meat, and special labels (e.g., green or organic chickens) were also collected.

### 2.2. Isolation and Identification of S. aureus

Isolation and identification of *S. aureus* were performed according to the EN ISO 6888-1 standard procedure of the International Organization for Standardization [24]. For pre-enrichment of bacteria, a 25 g portion from each piece of meat sample was chopped into very small fine pieces, homogenized with 225 mL of buffered peptone water, and incubated at 37 °C for 18–24 h. After pre-enrichment in buffered peptone water, 1 mL of the culture was mixed with 5 mL of nutrient broth and incubated for 24 h at 37 °C. Then, a loopful of culture broth was streaked onto Mannitol Salt Agar in duplicate and incubated at 37 °C for 24 h. Three presumptive *S. aureus* yellow color colonies with yellow zones from each selective agar plate were picked, and then subcultured to obtain a pure culture. Gram staining, catalase, and coagulase tests were performed with the pure culture. All presumptive *S. aureus* isolates were subjected to DNA extraction using the “boiling” method as described earlier [25]. A duplex PCR was carried out for the confirmation of *S. aureus* with two sets of genus- and species-specific primers. Primers used were Staph756F (5′-AACTCTGTTATTAGGGAAGAACA-3′) and Staph750R (5′-CCACCTTCCTCCGGTTTGTCACC-3′ for the amplification of 756 bp from *16S rRNA* gene, and Nuc450-F (5′-AGTATATAGTGCAACTTCAACTAAA-3′) and Nuc450-R (5′-ATCAGCGTTGTCTTCGCTCCAAATA-5′) for the amplification of 450 bp from *nuc* (thermonuclease) gene [26,27]. The PCR reaction mixture (25 μL) consisted of 12.5 μL of OneTaq Quick-Load PCR master mix with standard buffer (New England BioLabs Inc., Ipswich, MA, USA), 1.5 μL (15 pmol) each of forward and reverse primers, 2 μL of DNA template, and 4.5 μL of nuclease-free water. The thermal profile consisted of initial denaturation at 94 °C for 5 min, 30 cycles of separation at 94 °C for 1 min, primer annealing at 55 °C for 1 min, and extension at 72 °C for 2 min with a final elongation at 72 °C for 10 min. After amplification, PCR products were analyzed by gel electrophoresis on 1.5% UltraPure™ Agarose gel was stained with ethidium bromide (5 µg/mL) including a 100-bp DNA ladder (New England BioLabs Inc., Ipswich, MA, USA) which served as a molecular weight marker. The resulting band of PCR product was visualized under UV transilluminator and photographed. All PCR-confirmed *S. aureus* isolates were stored on nutrient broth containing 50% (*v*/*v*) glycerol at −20 °C for further study.

### 2.3. Antimicrobial Susceptibility Testing

The AMR profile of all *S. aureus* isolates was determined using the Kirby–Bauer disk diffusion method as described by the Clinical and Laboratory Standards Institute (CLSI) [28] with a panel of 38 antimicrobials representing 14 different antimicrobial classes. The antimicrobials commonly used for antimicrobial susceptibility testing include the fluoroquinolones (nalidixic acid (NA, 30 µg), ciprofloxacin (CIP, 5 µg), levofloxacin (LEV, 5 µg), norfloxacin (NX, 10 µg), ofloxacin (OFX, 5 µg), gatifloxacin (GAT, 5 µg), pefloxacin (PEF, 5 µg)), nonextended spectrum cephalosporins (first-generation cephalosporins: cephalexin (CL, 30 µg), cephradine (CE, 30 µg); second-generation cephalosporins: cefuroxime (CXM, 30 µg), cefaclor (CEC, 30 µg)), extended-spectrum cephalosporins (third-generation cephalosporins: cefotaxime (CTX, 30 µg), ceftriaxone (CRO, 30 µg), ceftazidime (CAZ, 30 µg), cefixime (CFM, 5 µg); fourth-generation cephalosporins: cefepime (FEP, 30 µg)), cephamycins (cefoxitin (FOX, 30 µg)), carbapenems (imipenem (IPM, 10 µg), meropenem (MEM, 10 µg)), tetracyclines (tetracycline (TE, 30 µg), oxytetracycline (OT, 30 µg), doxycycline (DO, 10 µg)), penicillins (ampicillin (AM, 10 µg), amoxycillin (AX, 10 µg), penicillin-G (P, 10 units), methicillin (MET, 5 µg), oxacillin (OX, 1 µg), cloxacillin (CX, 5 µg)), penicillins + β-lactamase inhibitors (amoxicillin-clavulanic acid (AMC, 30 µg)), aminoglycosides (gentamicin (CN, 10 µg), neomycin (N, 30 µg), tobramycin (TOB, 10 µg)), folate pathway inhibitors (trimethoprim-sulfamethoxazole (SXT, 25 µg)), lincosamide (clindamycin (DA, 2 µg)), glycopeptides and lipoglycopeptides (vancomycin (VA, 30 µg)), macrolides (azithromycin (AZM, 15 µg), erythromycin (E, 15 µg)), and polymyxins (colistin (CT)). For colistin, minimum inhibitory concentrations (MICs) were determined by broth microdilution method, according to the CLSI guidelines [28]. The interpretive category (susceptible, intermediate, and resistant) of each isolate was determined according to the CLSI guidelines [28], and in some cases, of European Committee on Antimicrobial Susceptibility Testing (EUCAST) [29]. Isolates resistant to at least one agent in three or more antimicrobial classes were defined as multidrug resistant (MDR) while isolates resistant to at least one agent in all but two or fewer antimicrobial classes were defined as possible extensively drug resistant (pXDR) [30].

### 2.4. Screening of Methicillin-Resistant S. aureus (MRSA)

Phenotypically, MRSA were detected by Cefoxitin Disk Diffusion method as per CLSI guidelines [28]. Briefly, for each isolate, a minimum of four to five *S. aureus* colonies isolated from an overnight growth were transferred to nutrient broth. Bacterial suspensions in nutrient broth at a density equivalent to a 0.5 McFarland standard was inoculated onto Mueller–Hinton agar plate in duplicate with cefoxitin (30 µg), oxacillin (1 µg), cloxacillin (5 µg), and methicillin (5 µg) disk. The plates were incubated at 37 °C for 24 h. The isolates of *S. aureus* that showed resistance to cefoxitin (≤21 mm zone diameter) were considered MRSA.

### 2.5. Detection of Methicillin Resistance Gene

A uniplex PCR targeting methicillin resistance gene (*mecA*) in *S. aureus* was standardized, and used in this study with specific primer as described earlier [27]. The sequence of the forward primer was MecA1 (5′-GTAGAAATGACTGAACGTCCGATAA-3′) and of the reverse primer was MecA2 (5′-CCAATTCCACATTGTTTCGGTCTAA-3′) for the amplification of 310 bp. Each PCR reaction mixture was constituted in a final reaction mixture of 25 µL made up of 12.5 µL PCR master mix (New England BioLabs Inc., Ipswich, MA, USA), 1.5 µL (15 pmol) each of forward and reverse primers, 7.5 µL of nuclease-free water, and 2 µL of DNA template. Amplification was performed by using a Veriti 96-well thermal cycler (Thermo Fisher Scientific) with this thermal profile: heating at 94 °C for 5 min, followed by 30 cycles of denaturation at 94 °C for 1 min, primer annealing at 55 °C for 1 min, extension at 72 °C for 2 min, and a final extension step for 10 min at 72 °C. Positive (*mecA* gene) and negative (sterile phosphate buffer saline) controls were included in each run of PCR. After amplification, PCR product was subjected to electrophoresis on 1.5% UltraPure™ agarose gel containing ethidium bromide (0.5 μg/mL). The resulting band of PCR product was examined under UV-transilluminator and photographed. A 100-bp molecular weight standard ladder was included on each run.

### 2.6. Data Analyses

Data were extracted, entered into a spreadsheet (Microsoft Excel) and transferred into SPSS software v22.0 (IBM Corp., Armonk, NY, USA) for statistical analysis. Descriptive statistics were used to compute the prevalence of *S. aureus* and resistance percentage. The significant differences in prevalence of *S. aureus* and resistance percentage among different brands, sampling area, chicken types, production types, and meat types were determined using chi-square test (Z-test for proportions) and Fisher’s exact test (wherever appropriate). The association between resistance phenotypes (outcome) and their corresponding methicillin resistance genes was analyzed by binary logistic regression. The level of significance was set at *p* ≤ 0.05. An UpSet plot was constructed to show the antimicrobial resistance pattern of methicillin-resistant *S. aureus* and methicillin-susceptible *S. aureus* by using online tools (https://asntech.shinyapps.io/intervene/ (accessed on 18 March 2021)).

## 3. Results

### 3.1. Prevalence and Distribution of Methicillin-Resistant S. aureus and Methicillin-Susceptible S. aureus

**Figure 1:**
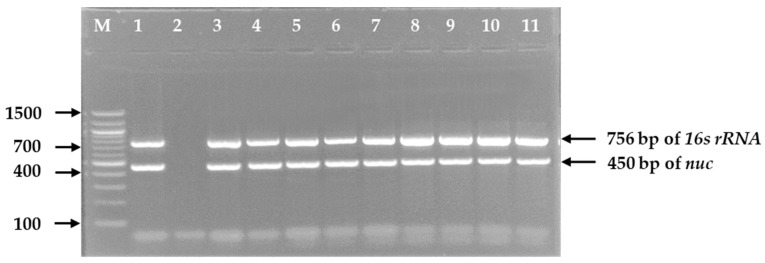
PCR amplified products of 756 bp of *16s rRNA* gene of *Staphylococcus* spp. and 450 bp of *nuc* gene of *Staphylococcus aureus* in 1.5% agarose gel electrophoresis and ethidium bromide staining. **Legends:** M = DNA marker (100 bp), Lane 1 = Positive control of *S. aureus*, Lane 2 = Negative control, Lane 3–11 = Tested *S. aureus* isolates.

Among 113 frozen chicken meat samples, the overall prevalence of *S. aureus* was 54.9% (*n* = 62) (Table 1). All isolates of *S. aureus* produced expected product size of 450 bp by PCR (Figure 1). Of the 62 *S. aureus* isolates, 23 (37.1%) were phenotypically identified as methicillin-resistant *S. aureus* (MRSA) based on cefoxitin disc diffusion test, and the remaining 39 (62.9%) isolates were methicillin-susceptible *S. aureus* (MSSA) (Table 1).

The distribution of MRSA and MSSA related to brands, divisions, chicken types, production types, and meat types is also summarized in Table 1. Among the nine brands, the prevalence of *S. aureus* from brand 7 (100%) was highest than those from brand 4 (11.3%). *S. aureus* was not recovered from frozen chicken meat of brand no. 8 and brand no. 9. The prevalence of MRSA among different brands varied from 36.8% to 50%, whereas the prevalence of MSSA varied from 50% to 100%. A significant difference was observed in the prevalence of MSSA among brands. Regarding division-wise distribution, the prevalence of MRSA was significantly higher in Chattogram division (66.7%) than Sylhet (33.3%) and Dhaka divisions (38.3%), while the highest prevalence of MSSA was found in Mymensingh and Rajshahi divisions (100%) than Chattogram division (33.3%). Chicken type-wise distribution revealed that there was significant difference in the prevalence of both MRSA and MSSA between broiler and cockerel chickens. MRSA prevalence in broiler and cockerel chickens were 45.2% and 20%, respectively, whereas the MSSA prevalence was 54.8% and 80%, respectively. When looking at the production type-wise distribution of MRSA and MSSA, there was no significant difference between production types. Meat type-wise distribution revealed that 100% of leg muscles were contaminated with MRSA, however, 73.9% of whole chicken pool sample were contaminated with MSSA.

### 3.2. Antimicrobial Resistance Pattern

Of the 62 *S. aureus* isolates, two isolates (3.2%) were possible extensively drug resistant (pXDR); it showed resistance to 11–12 of 14 antimicrobial classes. Regarding brand-wise distribution, it was observed that the pXDR *S. aureus* isolates were mostly observed in brands 1 and 3.

The overall MDR patterns of MRSA and MSSA are shown in Table 2. Among 62 *S. aureus* (both MRSA and MSSA) tested, all the isolates were MDR. It was revealed that more than half (52.2% for MRSA, and 53.8% for MSSA) of the isolates were resistant to 6–8 antimicrobial classes while 35.9% of MSSA isolates were resistant to 3–5 classes. Notably, 47.8% of MRSA and 10.3% of MSSA isolates were resistant to 9–12 classes. The distribution of MRSA and MSSA resistant to multiple antimicrobial classes showed that 100% isolates, recovered from brand 4 in case of MRSA, and brand 5 in case of MSSA, were resistant to 6–8 antimicrobial classes. Furthermore, 85.7% MRSA isolates from brand 1, and 100% MSSA isolates from brand 6 showed resistance to 9–12 and 3–5 antimicrobial classes, respectively. All the MRSA isolates from Sylhet division and 75% from Chattogram division were resistant to 9–12 classes of antimicrobials. On the other hand, 100% MSSA isolates, resistant to 3–5 and 6–8 antimicrobial classes, were observed in Sylhet and Rajshahi, and Chattogram divisions, respectively. Regarding chicken types, it was observed that 57.9% of MRSA isolates from broiler chicken meat, and 75% isolates from cockerel chicken meat were resistant to 6–8 and 9–12 antimicrobial classes, respectively. In case of MSSA, ≥ 50% of the isolates from broiler and cockerel chickens expressed resistance to 6–8 classes of antimicrobials. As for production types, 75% of MRSA and MSSA isolates from organically produced chickens were resistant to 6–8 and 3–5 antimicrobial classes, respectively, while more than 50% isolates of MRSA and MSSA from non-organically produced chickens were resistant to 9–12 and 6–8 classes, respectively. The meat type-wise analysis revealed that the highest resistance to 9–12 antimicrobial classes was observed in leg muscles (100%) in case of MRSA while the resistance to 6–8 classes was higher in wing muscles (80%) in case of MSSA.

**Figure 2:**
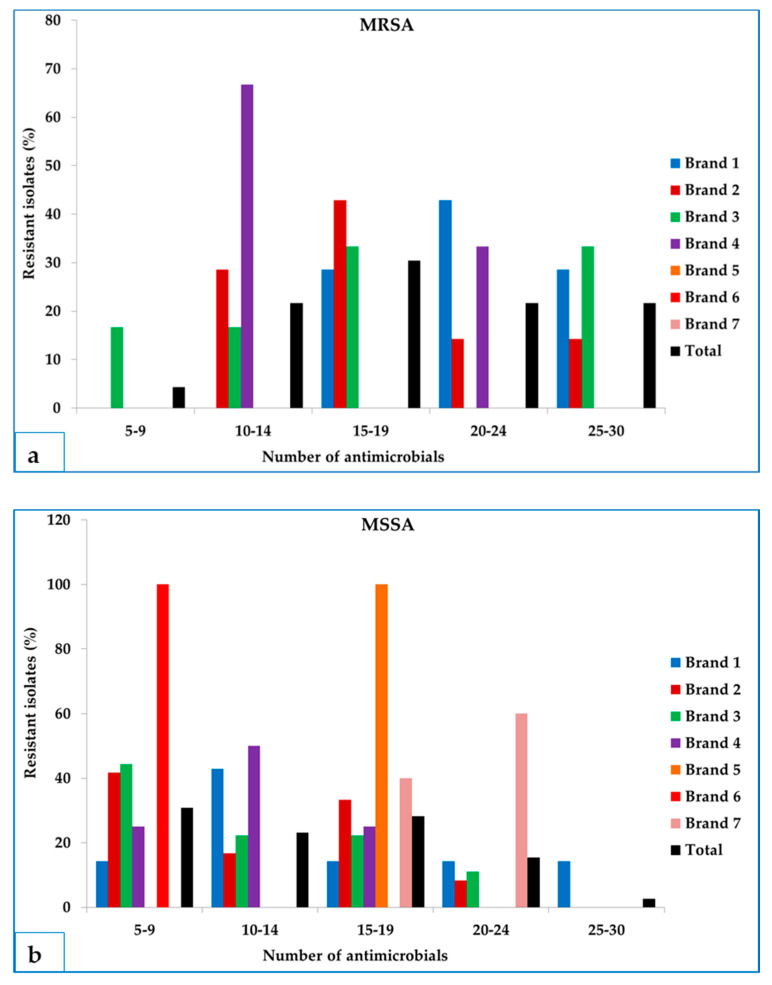
Brand-wise distribution of (**a**) MRSA and (**b**) MSSA isolates resistant to multiple antimicrobial agents isolated from frozen chicken meat.

Among 62 *S. aureus* isolates (both MRSA and MSSA), all the isolates were resistant to at least five, and up to 30 antimicrobial agents tested (Figure 2a,b). Regarding MRSA, 30.4% isolates were resistant to 15–19 antimicrobials, 21.7% isolates were resistant to 10–14 antimicrobials, and 4.3% isolates were resistant to 5–9 antimicrobials (Figure 2a). Notably, a noticeable number of MRSA isolates (10, 43.4%) demonstrated resistance to 20–30 antimicrobial agents (Figure 2a). For MSSA, resistance to 5–9 and 15–19 antimicrobials were observed in 30.8%, and 28.2% isolates, respectively (Figure 2b). Of note, seven (18%) isolates showed resistance to 20–30 antimicrobial agents (Figure 2b). Analysis by brands revealed that comparatively higher percentages of MRSA isolates demonstrated resistance to 10–14 antimicrobials in brand 4, followed by 15–19 antimicrobials in brand 2 and 20–24 antimicrobials in brand 1 than other brands (Figure 2a). On the contrary, the highest resistance to 5–9 and 15–19 antimicrobial agents was observed in brands 6 and 5, respectively, whereas resistance to 20–24 antimicrobial agents was higher in brand 7 (60%) in case of MSSA (Figure 2b).

**Figure 3:**
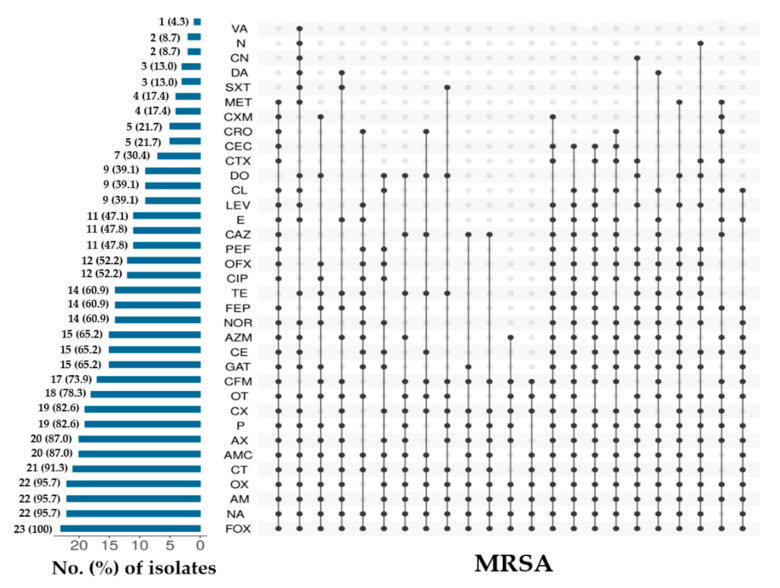
Individual antimicrobial resistance pattern of methicillin-resistant *S. aureus* (MRSA) isolated from frozen chicken meat.

**Figure 4:**
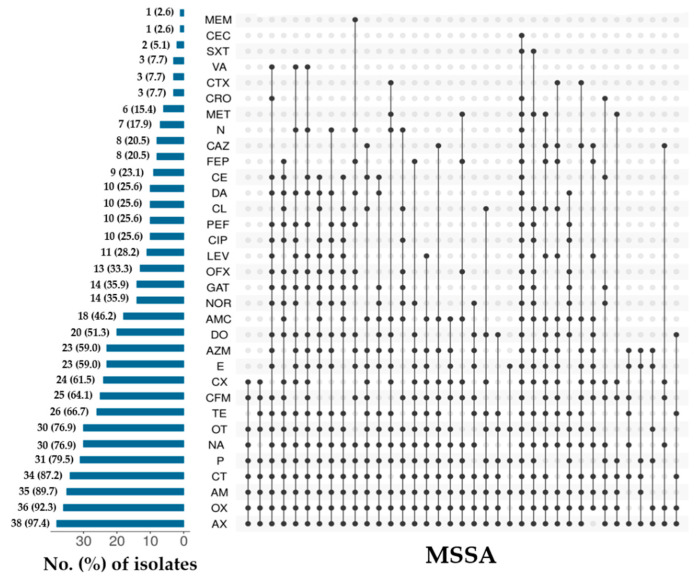
Individual antimicrobial resistance pattern of methicillin-susceptible *S. aureus* (MSSA) isolated from frozen chicken meat.

Overall individual antimicrobial resistance pattern of MRSA and MSSA is shown in Figure 3 and Figure 4. Among MRSA isolates, the resistance to cefoxitin (100%, *n* = 23), nalidixic acid, ampicillin and oxacillin (97.7%, *n* = 22), colistin (91.3%, *n* = 21), amoxicillin-clavulanic acid and amoxicillin (87%, *n* = 20), penicillin-G and cloxacillin (82.6%, *n* = 19), oxytetracycline (78.3%, *n* = 18), and cefixime (73.9%, *n* = 17) were found highest, whereas, resistance to vancomycin (4.3%, *n* = 1), and neomycin and gentamicin (8.7%, *n* = 2) were observed lowest (Figure 3). For MSSA, the highest individual AMR was observed against amoxicillin (97.4%, *n* = 38), oxacillin (92.3%, *n* = 36), ampicillin (89.7%, *n* = 35), colistin (87.2%, *n* = 34), penicillin-G (79.5%, *n* = 31), and nalidixic acid and oxytetracycline (76.9%, *n* = 30). Low resistance was observed to meropenem and cefaclor (2.6%, *n* = 1), and trimethoprim-sulfamethoxazole (5.1%, *n* = 2) (Figure 4). When looking at the AMR patterns, 23 different resistance patterns were observed for MRSA isolates, and 37 different resistance patterns were observed for MSSA isolates (Figure 3 and Figure 4). The most frequent AMR patterns observed among the MSSA isolates were AX-OX-AM-CT-P-NA-OT-CFM-CX (*n* = 2) and AX-OX-AM-CT-P-OT-TE-CFM-CX (*n* = 2) (Figure 4).

### 3.3. Prevalence of mecA Gene

**Figure 5:**
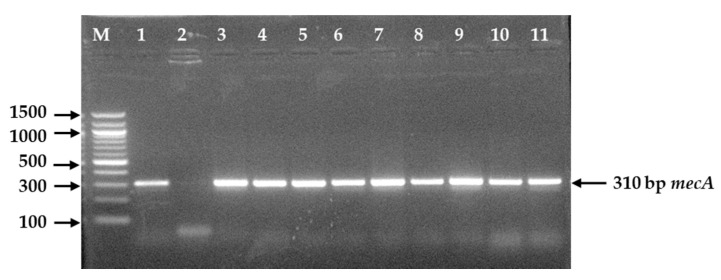
PCR amplified product of 310 bp from *mecA* gene of MRSA and MSSA following 1.5% agarose gel electrophoresis and ethidium bromide staining. **Legends:** M = DNA marker (100 bp), Lane 1 = Positive control of *mecA* gene, Lane 2 = Negative control, Lane 3–11 = Positive for *mecA* gene.

Among 23 MRSA and 39 MSSA, 10 (43.5%) and 17 (43.6%) isolates, respectively, were positive for *mecA* gene as they generated an expected size of 310 bp on amplification (Figure 5).

### 3.4. Phenotypic and Genotypic Association of Antimicrobial Resistance

In case of MRSA, among the seven isolates resistant to cefotaxime, six isolates (85.7%) carried the mecA gene (Table 3). For the 15 isolates resistant to gatifloxacin and azithromycin, the mecA gene was detected in nine isolates (60%). Eight (8/12, 66.7%) of the ciprofloxacin and ofloxacin resistant isolates also carried the mecA gene. However, these phenotypes were positively associated (OR > 1) with the presence of the mecA gene. For MSSA, a certain percentage of isolates (10/31, 32.3%) resistant to penicillin-G harbored the *mecA* gene (Table 3). Penicillin-G resistance was negatively associated with the presence of the *mecA* gene (OR = 0.1, *p* = 0.02).

## 4. Discussion

The present study provides the first comprehensive evidence on the extent and distribution of methicillin-resistant *S. aureus* (MRSA) and their AMR profile including methicillin resistance gene (*mecA*) isolated from frozen chicken meat in Bangladesh. Around 55% of frozen chicken meat samples were found positive for *S. aureus*, and of them 37% were identified as MRSA. The occurrence of MRSA in frozen chicken meat is consistent with earlier reports where 33.3% *S. aureus* isolates were identified as MRSA from processed raw meat samples in Bangladesh [23]. Compared with studies from other countries, a variable occurrence of MRSA (8.1–89%) in frozen chicken meat was reported in China and Egypt [10,11]. Variable occurrence may be due to differences in handling and management practices of frozen chicken meat samples and geographical location [10]. The present study demonstrated that the occurrence of MRSA as well as methicillin-susceptible *S. aureus* (MSSA) varied among different brands of frozen chicken meat. The highest distribution of MRSA was observed in the Chattogram division than Sylhet and Dhaka divisions of Bangladesh, which is in contrast with a previous report, where a comparatively lower percentage of MRSA contamination was observed in processed raw meat samples in Dhaka division of Bangladesh [23]. On the contrary, the occurrence of MSSA was higher in Mymensingh and Rajshahi divisions than Chattogram division. The chicken types (broiler vs. cockerel) had significant effect on the occurrence of MRSA and MSSA, however, production types (organic vs. non-organic) had no significant effects. Within the present study, a variable degree of contamination with MRSA was found among different types of meat samples. Contamination of frozen chicken meat with high occurrence of MRSA could be attributed to poor hygienic practices of meat handlers, and cross-contamination during slaughtering, or processing and packaging of chicken meat [10]. In spite of the fact that the nasal area is viewed as the primary site of colonization with *S. aureus*, these organisms are also present in the gastrointestinal tract of chickens [31]. During slaughtering, carcasses may get contaminated by the contents of the intestinal tract, from the slaughtering environment or even by MRSA-infected handlers who have direct contact with carcasses or meat [32].

Considering the obvious importance of *S. aureus* as foodborne pathogen, and the worldwide emergence of MDR in this foodborne bacterium, we screened the AMR profiles of *S. aureus* isolated from frozen chicken meat. An important finding of concern in this study is that all the isolates of MRSA and MSSA were MDR (resistant to three or more classes of antimicrobials), of which a significant proportion of the isolates were resistant to 6–8 and 9–12 antimicrobial classes. A previous report also documented that 100% MRSA isolates were MDR [23]. MDR MRSA from retail chicken meat were also reported from different parts of the world, 64.3% in Nigeria [6], 45.7% in India [33], and 44.4% in China [34]. The high occurrence of MDR MRSA and MSSA in frozen chicken meat was related with the type of brands, which might be due to brand level differences in production, handling, processing, and packaging including the type of antimicrobials used. Though all the branded supershops collected live broiler chickens from their contract farms, it was assumed that the farming practices in terms of biosecurity, hygiene, and use of antimicrobials were different among the farms. Of note, the current study additionally observed that 3.2% of *S. aureus* isolates were possible extensively drug-resistant (pXDR). A report from India depicts the development of an extensively drug-resistant *S. aureus* isolates in humans [35]. The high percentage of MDR and existence of pXDR may be due to the result from random chromosomal mutations and transfer of resistance genes via conjugation and transformation of the resistance transfer factor and resistance determinants or could be considered preliminary evidence suggesting the extensive use of antimicrobial agents in veterinary and medical practices for the control of bacterial diseases [7]. Reports from Bangladesh revealed that multiple antimicrobials are used indiscriminately in broiler chickens throughout the production cycle, which play a vital role in the emergence of antibiotic-resistant bacteria [36,37]. Another conceivable clarification is that the high percentage of MDR might be credited to the cross-contamination during slaughtering, handling and processing [23,32]. Therefore, strict regulations including good production practices, responsible use of antimicrobials, and hygienic measures in slaughtering and processing are essential to reduce the carcass contamination with drug-resistant *S. aureus*.

Clinical management of Staphylococcal disease depends on antimicrobial treatment which frequently fails due to forceful resistance of organisms to antimicrobials. We found that all the isolates in this study were resistant to at least five antimicrobial agents, and 43.4% isolates of MRSA, and 18% isolates of MSSA were resistant to 20–30 antimicrobial agents. There was a high percentage of antimicrobial-resistance among MRSA and MSSA, which is in disagreement with the previous report in China, in which 2.3% of *S. aureus* isolates in frozen chicken meat were resistant to 16–24 antimicrobials [11]. This may be due to the fact that the indiscriminate uses of antimicrobial agents in poultry production for therapy, prophylaxis and growth promotion, along with poor biosecurity and waste management systems accelerate the emergence of antimicrobial resistant pathogens, which may be implicated in foodborne antimicrobial resistant bacterial infection in humans [38].

Although antimicrobial use is a fundamental technique for control of *S. aureus* infection, especially for MRSA, attributable to its formidable ability to adapt to variable environmental conditions, this organism has an extraordinary capability to quickly get resistant to essentially all antimicrobials [39]. In the current study, we observed that all the MRSA isolates were resistant to cefoxitin. This prevalence was higher than the finding of earlier study in Bangladesh, where the author reported 33.3% isolates showed resistance to cefoxitin [23]. In the current study, we observed that all the MRSA isolates were resistant to cefoxitin. This prevalence was higher than the finding of earlier study in Bangladesh, where the author reported 33.3% isolates showed resistance to cefoxitin [11]. This prevalences were higher than the finding of earlier study in Central Africa [40]. *S. aureus* is well known to express the highest resistance to penicillin antimicrobial class, and penicillin resistance by Gram-positive bacteria has been reported since 1940 [39]. Nalidixic acid and oxytetracycline resistance were also commonly observed among MRSA and MSSA isolates. This prevalences were higher than the findings of earlier studies on frozen chicken meat in China and Central Africa [11,40]. This is not surprising, because oxytetracycline is one of the most commonly used antibiotics for treatment of infections in poultry and humans without basic programs and restrictive policies on the use of these antibiotics, therefore, very frequent occurrences of resistance in MRSA and MSSA are probably a consequence of this. On the other hand, quinolone resistance among *S. aureus* emerged quickly, very conspicuously among the methicillin-resistant strains. As a result, the capacity to use fluoroquinolones as antistaphylococcal agents was dramatically diminished. The explanations for the difference in rates of quinolone resistance between MRSA and MSSA strains are unclear. One causative issue is probably the antibiotic selective pressure, particularly within the hospital setting, leading to the choice and spread of the more antibiotic resistant MRSA strains [41]. Of note, in the present study, resistance to colistin, last-resort antimicrobials used for human therapy, was detected in 91% MRSA and 87% MSSA isolates, which might be due to the chromosomal mutations through amino acid substitution [42]. Furthermore, environmental, meat processing, and human hygiene related factors may have an effect on AMR of *S. aureus* isolated from frozen chicken meat samples. On the other hand, MRSA isolates in this study showed relatively low resistance to vancomycin, and MSSA isolates to meropenem and cefaclor, which may be due to the fact that these antimicrobials do not have veterinary preparations, and are not available for veterinary use, and also are not routinely used in clinical setting in Bangladesh.

MRSA has become a serious concern in food safety, and constitutes a major health care problem [6]. *S. aureus* isolates were designated as MRSA based on the presence of the methicillin resistance gene *mecA*. Interestingly, the frequency of the *mecA* gene in both MRSA and MSSA recovered from frozen chicken meat reached around 44% in the current study. Our result contrast sharply with data published in Egypt and China, reporting that 5.6% and 8.6%, respectively, of the *S. aureus* isolates from frozen chicken meat were *mecA* gene positive, suggesting that the presence of *mecA* gene is the principal evidence for the detection of MRSA isolates [10,11]. However, harboring *mecA* gene is not sufficient for methicillin resistance, because some *S. aureus* isolates that contain the *mecA* gene are still shown to be susceptible to methicillin [43].

This study also described the association between AMR phenotypes and presence of *mecA* gene in MRSA and MSSA isolated from frozen chicken meat samples. Among the 23 MRSA and 39 MSSA isolates, we observed that the occurrence of methicillin resistance gene (*mecA*) was found not only in phenotypically resistant isolates but also in phenotypically nonresistant isolates. The association between the AMR and the presence of *mecA* gene in MRSA and MSSA might be because of colocalization of resistance gene on the same genetic elements, and the possible coselection of many resistance genes by a single antimicrobial [44]. However, resistance genes can be linked to genetic elements, and the use of a particular antimicrobial can select for resistance not only to its own, but also potentially to a variety of other antimicrobials [44]. Since methicillin resistance gene alone is insufficient to confer resistance, further mechanisms are likely associated with the resistance phenotype of MRSA and MSSA strains.

It would be worthwhile if samples were taken from more outlets of various supershops, however, frozen chicken meat were purchased from most of the renowned supershops situated in five divisional megacities of Bangladesh; in this manner, the information is illustrative of the entire of Bangladesh.

## 5. Conclusions

To our knowledge, this is the first thorough study on the prevalence of MRSA in frozen chicken meat samples from different supershops of Bangladesh, and is the only study describing the presence of MDR, pXDR and *mecA* gene in frozen chicken meat. This study reported a relatively high prevalence of MRSA and high rates of MDR amongst the isolates, thus indicating the potential role of chicken meat in the dissemination of MDR MRSA strains in Bangladesh and highlighting the health risks for consumers. Therefore, periodic surveillance of antimicrobial resistance of these organisms in foods of animal origin in different geographical areas is needed. The high prevalence of MDR MRSA in frozen chicken meat samples emphasizes the need for better sanitary education of food handlers in hygienic practices focusing on their potential role as reservoirs and spreaders of MRSA. Hygienic measures should be taken to ensure the safety of food products, and a proper risk assessment should be conducted to further clarify the possible health hazard for consumers. Finally, the proper application of good manufacturing practice, good hygiene practice, and well-designed hazard analysis of a critical control point program in the slaughterhouses and processing units should be implemented.

## Figures and Tables

**Table 1:** Prevalence and distribution of MRSA and MSSA isolated from frozen chicken meat.

Variables	Total No. of Samples	No. (%) of *S. aureus*-Positive Isolates	MRSA, No. (%)	MSSA, No. (%)
**Brands**				
Brand 1	23	14 (60.9)	7 (50.0) ^a^	7 (50.0) ^a^
Brand 2	40	19 (47.5)	7 (36.8) ^a^	12 (63.2) ^a^
Brand 3	28	15 (53.6)	6 (40.0) ^a^	9 (60.0) ^a^
Brand 4	8	7 (11.3)	3 (42.9) ^a^	4 (57.1) ^a^
Brand 5	2	1 (50.0)	0	1 (100.0) ^b^
Brand 6	2	1 (50.0)	0	1 (100.0) ^b^
Brand 7	5	5 (100.0)	0	5 (100.0) ^b^
Brand 8	3	0	0	0
Brand 9	2	0	0	0
**Divisions**				
Dhaka	82	47 (57.3)	18 (38.3) ^a^	29 (61.7) ^a^
Chattogram	10	6 (60.0)	4 (66.7) ^b^	2 (33.3) ^c^
Sylhet	11	3 (27.3)	1 (33.3) ^a^	2 (66.7) ^a^
Mymensingh	5	5 (100.0)	0	5 (100.0) ^b^
Rajshahi	5	1 (20.0)	0	1 (100.0) ^b^
**Chicken types**				
Broiler	82	42 (51.2)	19 (45.2) ^a^	23 (54.8) ^b^
Cockerel	31	20 (64.5)	4 (20.0) ^b^	16 (80.0) ^a^
**Production types**				
Organic	10	8 (80.0)	4 (50.0) ^a^	4 (50.0) ^a^
Non-organic	103	54 (52.4)	19 (35.2) ^a^	35 (64.8) ^a^
**Meat types**				
Breast	27	13 (48.1)	5 (38.5) ^a,b^	8 (61.5) ^a^
Drumstick	30	14 (46.7)	5 (35.7) ^a,b^	9 (64.3) ^a^
Leg	3	3 (100.0)	3 (100.0) ^c^	0
Wing	19	9 (47.4)	4 (44.4) ^a^	5 (55.6) ^a^
Whole chicken pool sample	34	23 (67.6)	6 (26.1) ^b^	17 (73.9) ^a^
Total	113	62 (54.9)	23 (37.1)	39 (62.9)

^a,b,c^ Values in the same column for each variable with different superscripts differ significantly (*p* ≤ 0.05).

**Table 2:** Distribution of MRSA and MSSA isolates resistant to multiple classes of antimicrobials.

Variables	No. (%) of Isolates Resistant to Multiple Antimicrobial Classes					
	MRSA			MSSA		
	3–5	6–8	9–12	3–5	6–8	9–12
**Brands**						
Brand 1	0	1 (14.3) ^b^	6 (85.7) ^a^	1 (14.3) ^b^	5 (71.4) ^a^	1 (14.3) ^b^
Brand 2	0	4 (57.1) ^b^	3 (42.9) ^b^	5 (41.7) ^c^	7 (58.3) ^a,b^	0
Brand 3	0	4 (66.7) ^b^	2 (33.3) ^b^	4 (44.4) ^c^	5 (55.6) ^a,b^	0
Brand 4	0	3 (100.0) ^a^	0	3 (75.0) ^a^	1 (25.0) ^b,c^	0
Brand 5	0	0	0	0	1 (100.0) ^d^	0
Brand 6	0	0	0	1 (100.0) ^a^	0	0
Brand 7	0	0	0	0	2 (40.0)^b^	3 (60.0)^a^
**Divisions**						
Dhaka	0	11 (61.1) ^a^	7 (38.9) ^b^	11 (37.9) ^b^	17 (58.6) ^b^	1 (3.4) ^b^
Chattogram	0	1 (25.0) ^b^	3 (75.0) ^a^	0	2 (100.0) ^a^	0
Sylhet	0	0	1 (100.0) ^a^	2 (100.0) ^a^	0	0
Mymensingh	0	0	0	0	2 (40.0) ^b^	3 (60.0) ^a^
Rajshahi	0	0	0	1 (100.0) ^a^	0	0
**Chicken types**						
Broiler	0	11 (57.9) ^a^	8 (42.1) ^b^	10 (43.5) ^a^	13 (56.5) ^a^	0
Cockerel	0	1 (25.0) ^b^	3 (75.0) ^a^	4 (25.0) ^b^	8 (50.0) ^a^	4 (25.0) ^a^
**Production types**						
Organic	0	3 (75.0) ^a^	1 (25.0) ^b^	3 (75.0) ^a^	1 (25.0) ^b^	0
Non-organic	0	9 (47.4) ^b^	10 (52.6) ^a^	11 (31.4) ^b^	20 (57.1) ^a^	4 (11.4) ^a^
**Meat types**						
Breast	0	3 (60.0) ^a,b^	2 (40.0) ^a,b^	4 (50.0) ^a^	4 (50.0) ^b^	0
Drumstick	0	3 (60.0) ^a,b^	2 (40.0) ^a,b^	4 (44.4) ^a^	5 (55.6) ^b^	0
Leg	0	0	3 (100.0) ^c^	0	0	0
Wing	0	3 (75.0) ^a^	1 (25.0) ^a^	1 (20.0) ^b^	4 (80.0) ^a^	0
Whole chicken	0	3 (50.0) ^b^	3 (50.0) ^b^	5 (29.4) ^ab^	8 (47.1) ^b^	4 (23.5) ^a^
Total	0	12 (52.2)	11 (47.8)	14 (35.9)	21 (53.8)	4 (10.3)

^a,b,c,d^ Values in the same column for each variable with different superscripts differ significantly (*p* ≤ 0.05); Samples from brands 8 and 9 were negative for *S. aureus*.

**Table 3:** Association between antimicrobial resistance phenotypes and methicillin resistance *mecA* gene in MRSA and MSSA isolated from frozen chicken meat.

Antimicrobials	N_P_	ARG	P+/G+	P+/G−	P−/G+	P−/G−	N_G_	OR	95% CI	*p*-Value ^a^
**MRSA**										
Cefotaxime	7	*mecA*	6	1	4	12	10	18.0	1.6–198.5	0.02
Norfloxacin	14	*mecA*	9	5	1	8	10	14.4	1.4–150.8	0.03
Ciprofloxacin	12	*mecA*	8	4	2	9	10	9.0	1.3–63.0	0.03
Gatifloxacin	15	*mecA*	9	6	1	7	10	10.5	1.0–108.6	0.05
Pefloxacin	11	*mecA*	8	3	2	10	10	13.3	1.8–100.1	0.01
Ofloxacin	12	*mecA*	8	4	2	9	10	9.0	1.3–63.0	0.03
Azithromycin	15	*mecA*	9	6	1	7	10	10.5	1.0–108.6	0.05
**MSSA**										
Penicillin-G	31	*mecA*	10	21	7	1	17	0.1	0.01–0.6	0.02

N_P_: No. of isolates expressing phenotypic resistance to the indicated antimicrobials. P+/G+: No. of phenotypically resistance isolates (P+) with resistance genes (G+) for antimicrobials identified. P+/G−: No. of phenotypically resistance isolates (P+) with no resistance genes (G−) for antimicrobials identified. P−/G+: No. of phenotypically susceptible isolates (P−) with resistance genes (G+) for antimicrobials identified. P−/G−: No. of phenotypically susceptible isolates (P−) with no resistance genes (G−) for antimicrobials identified. N_G_: No. of isolates carrying the indicated resistance gene. ^a^ Only statistically significant (*p* ≤ 0.05) associations are shown. ARGs = antimicrobial resistance genes; OR = odds ratio; CI = confidence interval.
